# Hemoglobin Expression in Nonerythroid Cells: Novel or Ubiquitous?

**DOI:** 10.1155/2014/803237

**Published:** 2014-11-05

**Authors:** Debarchana Saha, Mandar Patgaonkar, Ankit Shroff, Kanchana Ayyar, Tahir Bashir, K. V. R. Reddy

**Affiliations:** ^1^Division of Molecular Immunology and Microbiology (MIM), National Institute for Research in Reproductive Health (NIRRH), J.M. Street, Parel, Mumbai 400012, India; ^2^Department of Biological Sciences, Tata Institute of Fundamental Research (TIFR), Mumbai 400005, India

## Abstract

Hemoglobin (Hb) is a major protein involved in transport of oxygen (O_2_). Red blood cells (RBCs) contain maximum amount of Hb and because of their unique structure and plasticity they transport O_2_ to various tissues of the body at an optimal concentration. Recently, it has been reported that, apart from RBCs, Hb is also expressed by nonerythroid cells such as epithelial cells of different origin. The cells expressing Hb are from the tissues where maintenance of O_2_ homeostasis is of paramount importance. Hb expression has been observed in the epithelial cells from human tissues including lungs, neurons, retina, and endometrium. Our group has recently demonstrated that Hb is expressed by the cervicovaginal epithelial cells. We further showed that, apart from maintaining O_2_ homeostasis, Hb and the peptides derived from it play an indispensable role in the protection of vaginal epithelium by exhibiting antimicrobial activity. In this review, we discuss the significance of Hb expression in vaginal epithelial cells and its role in the recognition of pathogens thereby reducing the risk and/or severity of inflammation and/or infections and the possible mechanism by which Hb exhibits antimicrobial and antioxidative functions.

## 1. Introduction

Hemoglobin (Hb) was accidentally discovered by Hünefeld in 1840 in samples of earthworm blood held under two glass slides. He occasionally found small plate-like crystals in desiccated swine or human blood samples [1, 2]. These crystals were later named as “Haemoglobin” by Hoppe-Seyler in 1864 [3]. Around 1870, Claude Bernard discovered its role as oxygen carrier [4, 5]. However the discovery of the detailed three-dimensional structure of Hb by X-ray crystallography is credited to Perutz et al. [6] for which he was awarded the Nobel Prize (1962) in chemistry along with Sir John Kendrew.

Hb is an iron-containing oxygen-transport metalloprotein found majorly in the red blood cells (RBCs) of all vertebrates [7] except in the fish family Channichthyidae [8] as well as in some invertebrates. The main function of Hb in mammals is to transport oxygen (O_2_) from the lungs to various tissues of the body, but it is also known to interact with three other gases such as carbon dioxide (CO_2_), carbon monoxide (CO), and nitric oxide (NO).

Hb is a tetramer made up of two *α*-globin chains and two *β*-globin chains which are encoded by two genes located on chromosomes 16 and 11, respectively. The *β*-globin gene cluster is packaged into inactive heterochromatin in nonerythroid cells, whereas the Hb-*α* genes are imbedded in open chromatin conformations in all cell types [9]. Each of these chains has a heme moiety attached; thus individual chains can transport oxygen. Under physiological conditions it is involved in transport of oxygen from respiratory organs like lungs to various tissues through red blood cells (RBCs).

The old belief that Hb and its derived peptides are expressed only by the cells of the erythroid lineage has recently been challenged by many groups who have proved that Hb expression is much more versatile than previously assumed [10]. Recent advances in Hb research have shown its expression in a wide variety of nonerythroid cells ranging between macrophages [11], alveolar epithelial cells [12, 13], lungs [14], retinal pigment epithelial cells [15], mesangial cells of rat and human [16], hepatocytes [17], neuronal/glial cells [18], endometrial cells [19], and cervical cells [20].

Studies from our laboratory revealed that Hb is expressed by epithelial cells from cervicovaginal tissue of rabbit [21]. We have also proved that, apart from maintaining the oxygen homeostasis, Hb and peptides derived from it play an important role in the protection of the vaginal epithelium by exhibiting antimicrobial properties.

### 1.1. Macrophages

Non erythroid expression of Hb was first reported in murine macrophages by Liu et al. [11]. On treating with LPS and interferon (IFN-4 *γ*), they found the activation and expression of only Hb-*β* minor gene in the stimulated macrophage RAW264.7 cells. The induction of *β*-globin was shown from 3.5 hrs posttreatment and it lasted for at least 24 hrs. Globin genes have been known to be activated in cell lines transformed by viruses [22, 23]. The authors thus speculated that maybe the Hb expression in these macrophages was due to the virus transformation. To rule this out, they repeated their study in peritoneal macrophages collected from BALB/c mice and confirmed the expression of Hb-*β* minor gene in the stimulated cells. The authors hypothesized that the transcriptional machinery involved in the expression of Hb in the activated macrophages is different and may involve erythroid-specific transcription activators like erythroid Krüppel-like factor (EKLF) or erythropoiesis-inducing transcription factor hypoxia inducible factor 1 (HIF-1), which may play a role in the transcription of globin genes in activated macrophages in response to hypoxia- or oxidant-related stress. They further speculated that Hb has a different functional role in nonerythroid cells by metabolizing NO and giving protection against nitrosative stress. However, they did not find any relationship between the Hb expression and NO decay rate in both stimulated and nonstimulated cells. So they concluded that monomeric globin might function in facilitating oxygen transfer to sites where its extensive use is required or have an alternative enzymatic function or help as O_2_/NO sensor. Though the authors have shown Hb expression, they have only found Hb-*β* minor gene and that too only in the stimulated macrophages.

### 1.2. Alveolar Epithelial Cells

Bhaskaran et al. [13] reported significant expression of Hb-*α* and Hb-*β* genes in type II alveolar epithelial cells (AECs) while characterizing differentially regulated genes in type I AECs and type II AECs using DNA microarray analysis. Type II AECs are in close proximity to the environmental oxygen and hence authors have proposed that Hb might be involved in sensing the oxygen concentrations, facilitation of oxygen transport across air-blood barrier, and scavenging oxygen/nitrogen to reduce oxidative/nitrosative stress. Newton et al. [12] extended these studies on human alveolar epithelial cells and demonstrated the presence of Hb subunits in ATII adenocarcinoma cells line (ATII cells), Clara-like adenocarcinoma cells (H441), and mouse alveolar epithelial cells (MLE-15). However, they have not determined the presence of heme prosthetic group in these cells. They propose that individual globin chains might have functional roles in these cells and Hb might be involved in pathologies associated with respiratory diseases.

Idiopathic pulmonary fibrosis (IPF) and chronic obstructive lung disease (COPD) are the major lung associated pathologies. Ishikawa et al. [14] characterized the proteomes of lung tissues taken from the normal individuals and individuals affected with these two pathologies to identify biomarkers for better prognosis of these diseases. They found that Hb levels are affected severely in the lung epithelium in patients suffering from IPF; however the levels remained unaffected in individuals with COPD. The reduced Hb levels in IPF cases were due to modification of thiol group in cysteine residue (C_105_) of Hb-*α* monomer. This residue is involved in complex formation between two Hb-*α* monomers which is essential for optimal function. The modification of this residue in Hb-*α* due to oxidative stress in IPF individuals does not allow the formation of Hb complexes. The authors have shown presence of Hb in tetrameric form in bronchoalveolar lavage fluid (BALF) and sputum and have shown that Hb is secreted into these fluids from lung tissues.

Recently Grek et al. [24] have shown enhanced Hb-*α* and Hb-*β* expression in ATII cells and MLE-15 cells after induction with hypoxia, where they have clearly determined the relation between oxidative stress and Hb expression. In this study, authors have reported that regulation of Hb synthesis in the ATII cells occurs in similar fashion as it takes place in erythroid precursor cells. However, alveolar macrophages did not show expression of any of the genes involved in hematopoietic development. They have determined that hypoxia inducible factors (HIFs) play essential role in Hb expression in these cells. In this study these authors have proved that the transcription factor GATA-1 is required for expression of Hb genes in ATII cells. Long term knockdown of GATA-1 with shRNA construct showed up to 70% reduction in Hb expression at both transcript and protein levels. The authors conclude that HIFs play crucial role in maintaining the homeostasis in lungs where fluctuations in O_2_ concentrations may lead to detrimental effects.

### 1.3. Retinal Pigment Epithelial Cells

Retinal epithelial cells, especially photoreceptors, require high amounts of oxygen which is supplied by choroidal circulation via diffusion [25]. Tezel et al. [15] were intrigued about the proper functioning of the photoreceptors in cases of systemic hypoxia, where inner retinal neurons failed to work normally. They studied the proteome of retinal epithelial cells and showed that its major constituent is Hb and it compensates their need for excess oxygen. The authors further proved that Hb is secreted from retinal epithelial cells at the rate of 1.9 ± 1.2 attomoles per cell per hour. Electron microscope studies reveal that the secretion of Hb* in vivo* is through basolateral membrane. These new findings would be useful to understand the oxygen transport to outer retina and pathogenesis of age related macular degradation.

### 1.4. Mesangial Cells

Kidney is very sensitive to the oxygen changes due to oxygen diffusion between arterial and venous blood vessels. In a study to investigate pathogenesis of chronically hypoxic kidneys, Nishi et al. [16] observed increased expression of Hb in rat kidney cells. The authors found expression of Hb in the glomerular mesangial cells. The significance of Hb expression in these cells was determined by* in vitro* overexpression studies. Primary rat mesangial cells were cultured and stimulated with hydrogen peroxide (H_2_O_2_) to induce chronic hypoxia, which resulted in significant increase of Hb-*α* and Hb-*β* transcripts. When these cells were transfected with vectors carrying Hb-*α* and Hb-*β* genes, overexpression of these proteins was observed. These transfected cells were then stimulated with 1000 *μ*M H_2_O_2_ to analyze if the Hb expression has antioxidative properties. It was observed that intracellular generation of reactive oxygen species (ROS) was inhibited significantly in the cells expressing excess of Hb as determined by immunofluorescence studies. Lactate dehydrogenase (LDH) assay showed increase in the viability of Hb overexpressing cells compared to normal cells in the presence of 1000 *μ*M H_2_O_2_.

### 1.5. Hepatocytes

Liu et al. [17] have studied the pathogenesis of nonalcoholic steatohepatitis (NASH). While analyzing microarray data to identify differentially expressed genes in NASH they found that free Hb-*α* and Hb-*β* subunits in serum were significantly increased from normal controls to steatosis and to NASH. They hypothesized that oxidative stress upregulates Hb expression and Hb overexpression suppresses oxidative stress in HepG2 cells suggesting that maybe Hb plays a protective role in NASH. They confirmed the expression of Hb in NASH liver biopsies and HepG2 cells. NASH biopsies showed Hb-*α*1 and Hb-*β* protein expression in the cytoplasm of the hepatocytes and there was a 6.7-fold increase and a 7.9-fold increase in the relative copy numbers respectively. In HepG2, Hb-*α*1 transcript expression was about 17-fold higher than Hb-*β* transcript; however they were unable to detect Hb-*β* protein in HepG2 cells.

Overexpressing Hb-*α* and Hb-*β* in HepG2 cells and treating them with H_2_O_2_ showed significant reduction of oxidative stress in H_2_O_2_ treated Hb overexpressing cells, compared to H_2_O_2_ treated control cells. They speculated that increased Hb in NASH was due to the induction of erythropoiesis. But the absence of significant increase in the expression of erythrocyte specific genes such as delta aminolevulinate synthase 2 (ALAS2), glycophorin A (GYPA), *α*- and *β*-spectrin (SPTA, SPTB), and hematopoietic transcription factor GATA1 raised the possibility of a different mechanism other than erythropoiesis. They suggested that transcription factor, nuclear factor- (erythroid-derived 2-) like 2 (Nrf2), which is known to be a crucial mediator of oxidative stress, might be involved in the induction of Hb expression in these cells.

### 1.6. Neuronal Cells

Biagioli et al. [18] while studying gene expression profiles found the presence of *α*- and *β*-chain transcripts of Hb in the subpopulation of dopamine (DA) neurons, cortical and hippocampal astrocytes, and all mature oligodendrocytes of mouse brain. Gene expression analysis of MN9D cells stably transfected with Hb-*α* and Hb-*β* showed that Hb expression acts on the genes responsible for O_2_ homeostasis. So, they suggested that Hb may act as an oxygen reservoir for anoxic and hypoxic conditions and control mitochondrial function. Thus, Hb may play a key role in the normal physiology of the brain and neurodegenerative diseases.

Russo et al. [26] further extended their study and showed that Hb is present as *α*_2_ *β*_2_ tetramer when overexpressed in dopaminergic MN9D cell line. They suggested that neuronal Hb may retain some of the biochemical activities and biological functions of Hb of erythroid lineage laying down the foundation for a better understanding of its role in brain physiology and in neurodegenerative diseases. Schelshorn et al. [27] demonstrated Hb expression in rat and mice neurons. They showed Hb expression by immunostaining in only the cerebral cortex, hippocampus, striatum, and cerebellum of the mouse brain, but not in astrocytes and oligodendrocytes, which is contradictory to what Biogioli group [18] had reported. Overexpressing Hb in the E18 cortical neurons cultured from rat brain showed better oxygenation when subjected to hypoxia. They also found that erythropoietin (EPO) induces Hb expression in these neurons. Therefore, they hypothesized that hypoxia induces production of growth factor EPO which in turn stimulates Hb expression in the mouse brain and thus Hb may play a role in improving the oxygen situation. Also, they were the first to report the presence of total Hb along with its prosthetic group, heme. They showed the presence of heme indirectly by showing an 8.8-fold increase in the expression of ALAS which is a key enzyme in heme synthesis, after EPO injection.

Recently, Richter et al. [28] showed Hb-*α*2 and Hb-*β* expression in the nigral, cortical, and striatal neurons from rat brains and striatal neurons in mice. Immunostaining showed presence of Hb-*α* chains in the cortex, basal ganglia structures, hippocampus, and hypothalamus and Hb-*β* chains in cortical and thalamic dendrites, hippocampal cells, and substantia nigra pars reticulata of rat and human brain. Systemic treatment of rats with low doses of rotenone (a mitochondrial toxin) showed downregulation of Hb-*α*2 and Hb-*β* expression in the dopaminergic neurons of the SNC as well as in cortical pyramidal neurons and in GABAergic projection neurons of the striatum. Treatment with rotenone has been shown to result in inhibition of the mitochondrial complex I and elevation of oxidative stress [29]. Heme is known to be a strong activator of globin-chain transcription [30] whose synthesis takes place in the mitochondria. Also, Hb gene expression in erythroid cells is known to be dependent on the activity of the *α*-subunit of hypoxia-inducible factor 1 (HIF-1A) [31]. Thus, they attributed the downregulation of Hb expression to the reduced production of heme or inactivation of HIF-1A. They also concluded that maybe Hb plays a protective role against oxidative and nitrosative stress by binding to NO, since NO is the strongest known ligand of the ferrous heme iron of Hb, with a higher affinity than oxygen. Heme is known to be a strong activator of globin-chain transcription whose synthesis takes place in the mitochondria. Also Hb gene expression in erythroid cells is dependent on the activity of the *α*-subunit of HIF-1A.

### 1.7. Endometrial Cells

Oxygen homeostasis in endometrium is crucial during earliest stages of embryo development; fluctuations in the oxygen concentrations during 1st trimester may lead to miscarriages by disruptions of villous tissue [32]. Dassen et al. [19] have reported expression of Hb in endometrial epithelial cells at transcript as well as protein levels. These authors observed in this study that Hb expression in endometrium is not regulated by hormones. Immunolocalization studies with pan-Hb antibody revealed that Hb is expressed by epithelial cells as well as endothelial cells. Stromal cells also showed presence of Hb. In this study, the expression of cytoprotective enzyme, hemeoxygenase-1 (HO-1), was observed in the epithelial cells of endometrium. HO-1 is an enzyme that catalyzes degradation of heme moieties to biliverdin, iron, and CO. Heme is synthesized by all nucleated cells and its involvement in the metabolic reactions depends on the protein with which it binds as a prosthetic group. In case of Hb, heme has iron molecule that binds with oxygen molecule and thus transports oxygen in the system. However excess of free heme moieties is toxic to the cells: they carry out various oxidative reactions and protein aggregations and also damage DNA due to oxidations. This toxic response of heme is neutralized by HO-1 [33]. The presence of Hb and HO-1 enzyme in the endometrial tissue signifies the importance of these molecules in the tissue to regulate heme, iron, and oxygen concentrations.

### 1.8. Cervicovaginal Epithelial Cells

Oxidative stress is caused by an imbalance between the formation of active oxygen metabolites and the rate at which they are scavenged, and it has been associated with the pathogenesis and complications of several diseases, including cancer. The overproduction or inadequate removal of ROS such as H_2_O_2_, hydroxyl radicals, and superoxide anion is associated with cancer [17].

Li et al. [20] have reported the expression of Hb in both normal and cancerous human cervical tissue. Immunohistochemistry studies showed diffused cytoplasmic staining. On carrying out microarray, they found increased expression of both Hb-*α*1 and Hb-*β* in the patient tissue sample as compared to the normal. However they did not find any increase in the expression of transcription factors for erythroid differentiation and erythroid cell markers or in the other Hb genes such as Hb-*δ*, Hb-*ε*, Hb-*γ*, Hb-*θ*1, and Hb-*ζ*. They further confirmed these findings by qRT-PCR and immunostaining. Expression of Hb was also confirmed in the cancerous cell lines such as SiHa and CaSki as well as in human embryonic kidney 293 cells (HEK293) by qRT-PCR. They found the expression of Hb-*α*1 to be 9.6-fold higher than that of Hb-*β*. Hb expression was also found to be upregulated in the presence of H_2_O_2_ in HEK293, SiHa, and CaSki cell lines. Transient transfection of SiHa and CaSki cells overexpressing Hb-*α*1 and Hb-*β* showed reduced intracellular ROS and also improved cell viability in the presence of H_2_O_2_. The authors thus hypothesized that Hb is part of the endogenous antioxidant defense system and protects cells against oxidative damage in cervical cancer patients.

**Figure 1:**
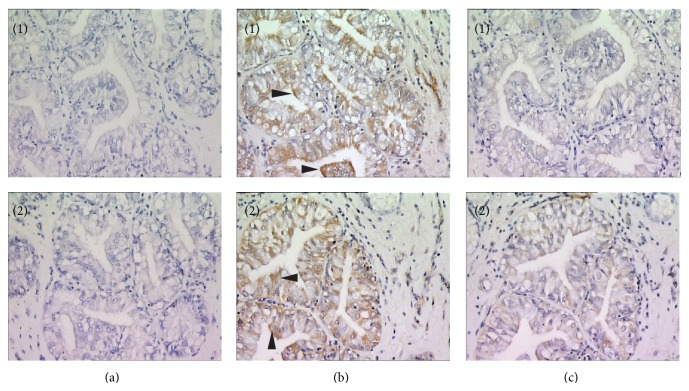
Immunohistochemical localization of RVFHb*α*P in rabbit vaginal tissue. RVFHb*α*P localized in glandular epithelium. Upper panel shows RVFHb*α*P localization and lower panel shows Hb localization. (a1) Negative control, (b1) anti-RVFHb*α*P antibody treated, and (c1) specificity control (10 *μ*g RVFHb*α*P + anti-RVFHb*α*P antibody). (a2) Negative control, (b2) anti-Hb antibody staining, and (c2) specificity control (10 *μ*g Hb + anti-Hb antibody). Each figure is the representative from three different experiments performed on three different days (magnification ×40; arrow indicates expression of RVFHb*α*P); Institute Animal Ethics Committee number (IAEC no. 1/2007).

**Figure 2:**
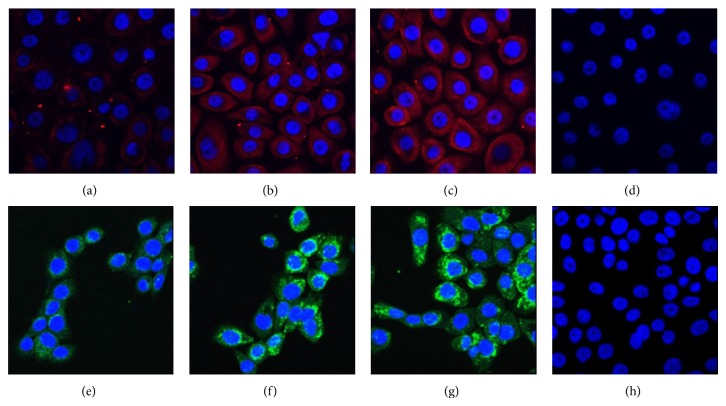
Indirect immunofluorescence localization of Hb-*α* and Hb-*β* subunits in cervicovaginal epithelial cells. The upper panel shows the expression of Hb-*α* (red) in VK2/E6E7 cells, control (a), H_2_O_2_ treated (b), LPS treated (c), and negative control (d). The lower panel shows the expression of Hb-*β* (green) in VK2/E6E7 cells, control (e), H_2_O_2_ treated (f), LPS treated (g), and negative control (h). Both subunits were found to be present in the cytoplasm of these cells. Nucleus was stained with DAPI (blue). The figure shown is the representative pictures from three independent experiments (magnification ×63).

Vaginal inflammation is linked to oxidative stress. Recent studies from our laboratory revealed the expression of Hb in cervicovaginal epithelial cells in rabbits. In one of the studies, we identified Hb subunits in the antimicrobial fraction of rabbit vaginal lavage. Further a peptide, RVFHb*α*P, was designed from Hb-*α* subunit which has shown antimicrobial activity [21]. The peptide showed activity against various pathogenic strains of microbes as well as HIV. The peptide has LPS binding activity and thus neutralizes the inflammatory effects caused by LPS [34]. In order to evaluate the localization of Hb in cervicovaginal tissue we used anti-RVFHb*α*P antibodies where it was observed that Hb-*α* was localized in the glandular epithelial cells. This expression was further confirmed by commercially available antibodies against whole Hb molecule where similar expression was observed (Figure 1). Preincubated mixture of Hb antibody and 10 *μ*g Hb was used as a specificity control, where no or negligible localization was observed. In our subsequent studies, three cell lines derived from cervicovaginal tissue of human and immortalized with HPV E6E7 were used [35]. We observed the expression of both subunits Hb-*α* and Hb-*β* in these cells using commercially available antibodies against these proteins (Figure 2). The expression of Hb-*α* and Hb-*β* was also observed at transcript level using gene specific primers. Currently we are studying this interesting phenomenon in more detail. Our preliminary results indicate that the expression of Hb increases 2-3-fold when the cells are treated with physiological concentration (20 *μ*M) of hydrogen peroxide which is found in human vaginal fluid [36]. The expression also increased on treatment with LPS (10 *μ*g).

At some instances spontaneous oxidation of ferrous heme ion (Fe^+2^) to ferric iron (Fe^+3^) occurs after attachment of oxygen leading to generation of superoxide radicals (O_2_^∙−^) and H_2_O_2_, which exerts oxidative stress to the host. Antioxidative enzymes like catalase and superoxide dismutase produced by RBCs carry out breakdown of superoxide radicals and H_2_O_2_ and also reduce Fe^+3^ to Fe^+2^ which can bind again to oxygen atom. Although approximately 1–3% of total Hb remains in oxidized form and it is known as methemoglobin (metHb), cell-free Hb is always in oxidized form due to reaction of Hb with environmental oxygen. H_2_O_2_ is toxic to the cells and recently it has been shown that Hb also possesses antioxidative pseudoperoxidase activity. Hb in the ferrous state reacts with H_2_O_2_ resulting in the formation of oxoferryl Hb (Hb-Fe^+4^=O). This protein-oxygen complex reacts further with H_2_O_2_ and leads to autooxidation of Hb leading to formation of ferric Hb or methemoglobin. This reaction leads to extensive cross-linking between the globin chains and oxidation of cysteine residues in *β* globin protein, which further leads to heme degradation [37]. This reaction can neutralize significant amounts of reactive oxygen species and protect cells by the adverse effects of H_2_O_2_. Moreover, heme liberated during these reactions is reduced to biliverdin, iron, and CO, protecting the cells from heme toxicity.

Lower female reproductive tract (FRT) functions to protect inner vaginal mucosal epithelial structures from microorganisms. Cellular oxidative stress leading to acute and chronic inflammation contributes to the bacterial and viral infections. Cervicovaginal cells are constantly in contact with H_2_O_2_ secreted by various lactobacilli, inhabiting vagina as commensals. H_2_O_2_ is one of the strong oxidizing agents. Hb, as mentioned earlier, may thus be involved in reducing the oxidative stress caused by various stimuli. Also, at the vaginal mucosal surface, where the host physically interacts with a nonsterile environment, the ability to detect and contain invading pathogens is regularly tested.* Escherichia coli* which are Gram-negative bacteria are one of the major causative agents for infections in vaginal cells. Gram-negative bacteria are the predominant cause of clinical sepsis. A major constituent of their outer membrane is lipopolysaccharide (LPS) [38], which is a potent stimulator of the immune system. LPS molecules shed from the bacterial surface contribute to the pathology of Gram-negative bacteria sepsis [39]. During Gram-negative sepsis LPS, an endotoxin produced by Gram-negative bacteria, stimulates various proinflammatory cytokines and mediators [40].

As mentioned earlier, Hb is an LPS binding protein and the structure and biological activity of Hb are altered in the presence of LPS [41]. LPS can oxidize Hb and convert metHb, which facilitates iron release and generation of free radicles, a condition known to contribute to Hb related antibacterial activity. Recently it has also been shown that Hb can synergize with LPS and LTA during macrophage activation and enhance innate immune responses by inducing expression of cytokines and chemokines [42, 43]. We hypothesize that the expression of Hb in these cells increases due to inflammation and might be required to neutralize the cytotoxic effects of H_2_O_2_ and LPS. Our preliminary observations support this hypothesis and further studies to corroborate these results are ongoing.

## 2. Conclusions and Future Directions

It is known that the expression of the Hb-*α* and Hb-*β* genes is balanced and coordinated. That is, both genes are expressed at comparable levels in erythrocytes and both are reportedly silenced in nonerythroid cells [9, 44]. Then why are the Hb genes expressed in nonerythroid cells and why is there a difference between the expression level of *α* and *β* genes? Some studies have reported increase in expression of Hb-*α* as compared to Hb-*β* [12, 19, 20] while others have shown the expression levels to be similar. In contrast to these studies, Liu et al. [11] have shown an increase in Hb-*β* minor and complete absence of Hb-*α* subunit in the stimulated macrophages. Thus, Hb expression in nonerythrocytes may be cell type specific. It is also observed that Hb expression in nonerythrocytes varies under different conditions. For example, hemoglobin expression was observed to be upregulated in response to oxidative stress and is believed to alleviate it [16, 17, 20]. However, the molecular mechanism of this pathway is poorly understood. It is established that erythroid Hb plays an important role in oxygen transfer and transport. Similarly, in some nonerythroid cells, Hb expression was found to be upregulated in response to hypoxia [15, 24, 26]. It appears that, to compensate for the increased oxygen demand, Hb is expressed in these cells. Therefore, expression of Hb genes in nonerythroid cells under de novo and specific conditions needs to be investigated in detail.

It is known that cell-free Hb is cytotoxic in nature. Cell-free Hb molecules have intrinsic toxicity to tissues since harmful ROS is produced during autoxidation of oxyHb to metHb [45]. Then why is Hb produced in the nonerythroid cells if it is harmful? Maybe the amount of Hb produced in these nonerythrocytes is way below the amount present in RBCs and, thus, inefficient to cause cytotoxicity to the host cells.

As noted above, expression of Hb-*α* and Hb-*β* is not comparable in nonerythrocytes. To understand this disparity, various studies have analyzed the transcription factors involved in regulating the expression of these genes in nonerythrocytes. GATA-1 is an important transcription factor; it plays a major role in switching fetal hemoglobin to adult hemoglobin during erythropoiesis. Some studies have shown its expression in cells such as neural, alveolar, cervical, hepatocytes [17, 18, 20, 24], while Richter et al. have demonstrated its absence in neural cells [28]. Apart from GATA-1, transcription factors like NFE2, KLF1/EKLF, Hif1-*α*, and so forth are expressed* de novo* or after stimulation. This raises the question whether there are different regulatory mechanisms acting in different cell types. Hence, it is important to study the interplay of various transcription factors depending upon their specific functions in different nonerythroid cells. It would also be interesting to understand whether Hb expression is regulated by environmental stress and if its regulation is mediated by different transcription factors depending on the external stimuli.

While showing the expression of Hb at protein and transcript level, most authors have shown the presence of only the globin chains and not the prosthetic group, heme and/or complete Hb tetramer. An attempt in this direction was made by Schelshorn et al. [27]. They have shown the presence of heme through an indirect observation: increased transcription of ALAS, a key enzyme in heme synthesis. However, this may not necessarily mean that Heme is associated with globin chains since heme is synthesized in all human nucleated cells [46] (it is a component of myoglobin, cytochrome, catalase, etc.). Since it is now known that the expression of both the globins is not comparable in nonerythrocytes, Hb or its constituents may have different functional significance wherein only the globin monomers, not the entire Hb tetramer, are required. Further studies to elucidate the complete structure of Hb expressed in nonerythroid cells and whether it varies from the Hb found in the erythroid cells need further confirmation.

Till date, Hb expression has been found in different cells ranging from macrophages to alveolar, lungs, hepatocytes, mesangial, retinocytes, endometrium, cervix, vaginal, and neuronal cells. Maybe with further research we will see that Hb has more unique functions and is ubiquitously expressed in various other cell types of our body. Hence, further studies are required to elucidate the exact mechanism of Hb expression and its functions in nonerythroid cells.
